# Proteomic Analysis of Humoral Immune Components in Bronchoalveolar Lavage of Patients Infected or Colonized by *Aspergillus fumigatus*

**DOI:** 10.3389/fimmu.2021.677798

**Published:** 2021-05-26

**Authors:** Sarah Dellière, Magalie Duchateau, Sarah Sze Wah Wong, Quentin Giai Gianetto, Hélène Guegan, Mariette Matondo, Jean-Pierre Gangneux, Vishukumar Aimanianda

**Affiliations:** ^1^ Institut Pasteur, Molecular Mycology Unit, CNRS, Paris, France; ^2^ Université de Paris, Paris, France; ^3^ Department of Mycology & Parasitology, Hôpital Saint-Louis, Paris, France; ^4^ Institut Pasteur, Proteomics Platform, Mass Spectrometry for Biology Unit, CNRS, Paris, France; ^5^ Institut Pasteur, Bioinformatics and Biostatistics Hub, Computational Biology Department, CNRS, Paris, France; ^6^ University of Rennes, CHU Rennes, Inserm, EHESP, Irset (Institut de recherche en santé, environnement et travail) - UMR_S 1085, Rennes, France

**Keywords:** *Aspergillus fumigatus*, aspergillosis, bronchoalveolar lavage, proteomics, humoral immunity, complement system, pentraxin-3

## Abstract

Humoral immune components have been individually studied in the context of interaction of host with *Aspergillus fumigatus*, a major airborne fungal pathogen. However, a global view of the multitude and complex nature of humoral immune components is needed to bring new insight into host-*Aspergillus* interaction. Therefore, we undertook comparative proteomic analysis of the bronchoalveolar lavage fluid collected from individuals infected or colonized with *A. fumigatus* versus controls, to identify those alveolar humoral components affected upon *A. fumigatus* infection. Complement proteins C1q, C8 beta-chain, factor-H, ficolin-1, ficolin-2, mannan binding lectin serine peptidase 2, pentraxin-3 and the surfactant protein-D were identified as the major humoral immune components affected by *A. fumigatus* infection and colonization. Based on this observation, we hypothesize that crosstalk between these humoral components is essential during host-*Aspergillus* interaction giving new specific leads to study for better understanding the pathogenesis. Furthermore, the affected humoral components could be potential diagnostic markers of *A. fumigatus* infection or colonization.

## Introduction

*Aspergillus fumigatus*, an airborne fungal pathogen, causes a range of allergic entities to invasive and chronic infections in patients with immune disorders and/or underlying pulmonary dysfunctions (1). The morbidity and mortality rate due to aspergillosis, the infection caused by *A. fumigatus*, remains high, which could be partly because of our poor knowledge on immunobiology of this fungus.

Innate immunity is the essential determinant of anti-*Aspergillus* response, which includes cellular and humoral components (composed of complement system, collectins, antibodies, acute-phase proteins and antimicrobial peptides). Although the cellular immune system against *A. fumigatus* is well studied, the role of humoral immune system against this fungus is an underexplored and/or a disregarded field. A better understanding of humoral components specifically implicated may both serve to develop new therapeutic strategies and to identify candidate host humoral markers of aspergillosis (2). *A. fumigatus* conidia entering lung-alveoli firstly interact with humoral components. Conidial interaction with bronchoalveolar lavage fluid (BALF) or serum showed significant difference in the humoral immune components interacting and subsequent immune response (3). This justifies specifically the use of BALF in identifying major players of humoral immunity against *A. fumigatus*.

We, therefore, undertook comparative proteomic analysis of the BALF from *A. fumigatus* infected or colonized (*Aspergillus*+) hosts versus controls, to identify humoral components altered by the presence of this fungus. In the *A. fumigatus*+ BALF, specific humoral components were absent or exclusively present, and the most affected ones were the complement components, suggesting their potential association with and the role against *A. fumigatus* conidia entering lung-alveoli.

## Method

### Sample Preparation

BALF samples were collected for diagnostic purposes prior to any treatment. For culture, BALF samples (5-10 mL) were centrifuged, re-suspended pellet (200µL) was inoculated on two Sabouraud agar plates, incubated at 30°C and 37°C, respectively, for 8-days for macroscopic and microscopic examinations of the mold. For PCR targeting a 67-bp sequence of the *A. fumigatus* 28S rRNA gene, 1 mL BALF was centrifuged and DNA was extracted from the pellet, using the QIAamp^®^ DNA minikit (Qiagen). Galactomannan and anti-*Aspergillus* antibody detections were performed using the Platelia *Aspergillus* Ag and IgG kits (Bio-Rad), respectively. Cryopreserved aliquots of BALF were used for our study; characteristics of the individuals from whom BALF collected are detailed in **Supplementary Table S1**. This was a non-interventional study with no additional sampling or change in the sampling procedures, and data were completely anonymized. According to the French Health Public Law (CSP Art L 1121-1.1), such protocol is exempted from informed consent application. *Aspergillus+* BALF were classified as (i) invasive pulmonary aspergillosis (IPA) according to EORTC/MSG consensus criteria (4), (ii) chronic pulmonary aspergillosis (CPA) according to ESCMID/ERS guidelines (5) or (iii) colonization if classification criteria were not met. Finally, all BALF from infected and colonized patients (n=10) and all control BALF (n=10) were pooled as *Aspergillus*+ and control (*Aspergillus-*) groups, respectively; BALF from each individual were pooled in equal volume for a group. Protein concentrations in both pools were measured by Bradford assay and divided into three technical replicates for proteomics.

### BALF-Digestion

Respectively, control or *Aspergillus*+ BALF of 35 μg and 130 μg protein per replicate were diluted in 8 M urea, 100 mM Tris HCl pH 8.5 to a final urea concentration of 6 M, reduced with 5 mM Tris(2-carboxyethyl)-phosphine (TCEP) for 30 min and alkylated with 10 mM iodoacetamide (IAA) for 30 min at room temperature in dark, then incubated with Mass Spec Grade rLys-C (protease:protein ratio 1:35; Promega, Madison, USA) for 5 h at 30°C, diluted below 2 M urea with 100 mM Tris HCl pH 8.5 and incubated with Sequencing Grade Modified Trypsin (ratio 1:70; Promega, Madison, USA) overnight at 37°C. A second incubation with trypsin at a same ratio was performed (5 h at 37°C) to ensure complete digestion. The digestion was stopped by adding 5% formic acid (FA). Resulting peptides were desalted and concentrated on Sep-Pak C_18_-SPE cartridge (Waters, Milford, USA) according to manufacturer instructions. Peptides were eluted using 50% acetonitrile (ACN)-0.1% FA, lyophilized and kept at -80°C until further use.

### LC-MS Analysis

Purified peptides were analyzed on a Q Exactive Plus mass-spectrometer (Thermo Fisher Scientific, Bremen) coupled with an EASY-nLC 1200 chromatography system (Thermo Fisher Scientific). Peptide-digests (1 µg) were loaded and separated at 250 nL/min^-1^ on an in-house packed 50 cm nano-HPLC column (75 μm inner diameter) with C_18_ resin (1.9 μm particles, 100 Å pore size, Reprosil-Pur Basic C18-HD resin, GmbH, Ammerbuch-Entringen, Germany) equilibrated in 97% solvent-A (H_2_O, 0.1% FA) and 3% solvent-B (ACN, 0.1% FA). Peptides were eluted with a linear gradient from 3 to 22% solvent-B in 160 min, followed by a stepwise increase of solvent-B to 50% in 70 min and finally to 90% in another 5 min. Mass-spectra were acquired with a Top10 data-dependent acquisition mode, with a scan range set to 300–1,700 m/z and an AGC target value of 3x10^6^. The fragmentation of precursor ions was performed by HCD (NCE 27) at 17.5 K resolving power (at m/z 200) with an AGC target value of 1x10^6^ and a maximum injection time of 60 ms. Precursors with unknown charge state or charge state of <1 and >7 were excluded; dynamic exclusion was set to 35 s.

### Protein Identification and Quantification

All raw files were searched with MaxQuant (v.1.5.3.8) against the Uniprot *Homo sapiens* reference proteome (20,416 entries) concatenated with the Uniprot *Neosartorya fumigata* reference proteome (9,647 entries), the usual contaminants and the reversed sequences of all entries, using trypsin as specific enzyme with a maximum of 3 missed cleavages. Possible modifications included carbamidomethylation (Cys, fixed), oxidation (Met, variable) and N-ter acetylation (variable). The mass tolerance was set to 20 ppm for the first search then 6 ppm for the main search and 10 ppm for the MS/MS. Maximum peptide charge was set to seven. Five amino acids were required as minimum peptide length. The “match between runs” feature was applied for samples having the same experimental condition with a maximal retention time window of 0.7 min. One unique peptide to the protein group was required for the protein identification. A false discovery rate cut-off of 1% was applied at the peptide and protein levels.

The statistical analysis of the proteomics data was performed as described previously (6). Functional annotation of BALF proteomes was conducted using database for annotation, visualization, and integrated discovery (DAVID) v6.8 (7). Overrepresented functional categories among proteins enriched in each subject population were identified relative to total unique protein found in BALF from our study using a permutation-based false discovery rate analysis (FDR). Processes with at least three protein members and FDR <5% were deemed significant. Protein-protein interactions were analyzed and sketched with STRING database v11.0 (8).

## Results

**Figure 1:**
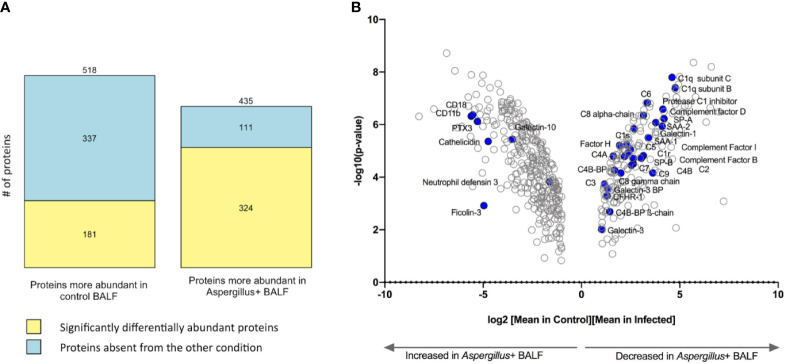
Differentially abundant proteins in *Aspergillus*+ bronchoalveolar lavage fluid (BALF) as compared to control BALF. **(A)** Number of proteins significantly differentially abundant (yellow) or absent from the other condition (in blue) and **(B)** Volcano plot showing 505 proteins with log2 (fold-change) >1 between control and *Aspergillus*+ conditions; humoral immune components majorly varying in the *Aspergillus*+ BALF are highlighted in blue.

A total of 1,177 proteins were identified in the BALF of all 20 samples (ten BALF samples each from controls and *Aspergillus*+ individuals). Three technical replicates showed strong correlation (**Supplementary Figure S1**). Totally, 953 proteins showed differential abundance of >2-folds in *Aspergillus*+ BALF compared to that of the control group (**Figure 1A**). The log2 fold-changes ≥1 and *p*-values of the quantitative analyses are depicted in a volcano-plot with humoral components of the innate immune system highlighted (**Figure 1B**).

**Figure 2:**
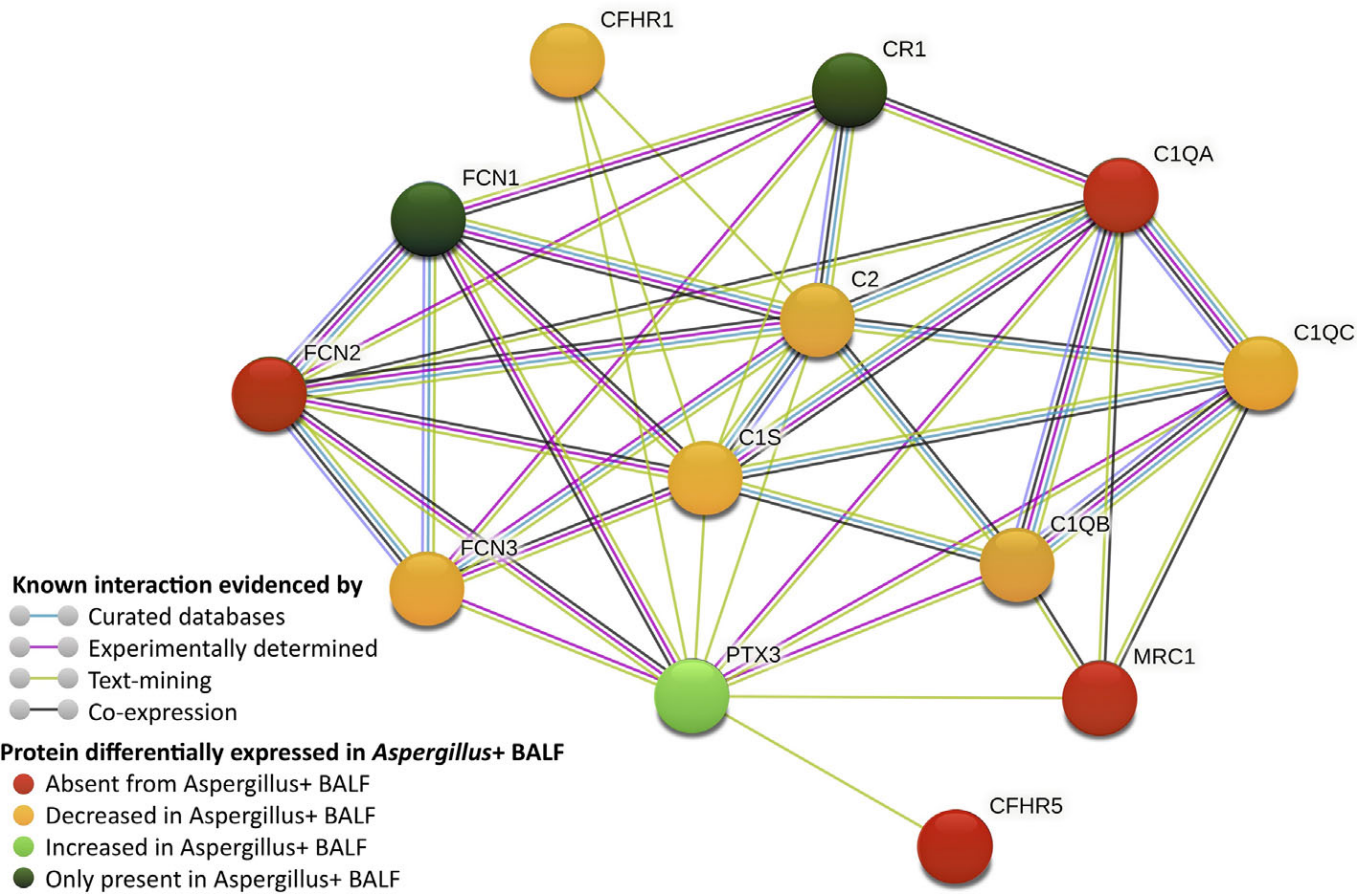
Network of complement components interacting with pentraxin-3 (PTX3), which are differentially expressed in *Aspergillus*+ BALF (generated using STRING database). CFHR, complement factor-H related protein; CR, complement receptor; FCN, ficolin; MRC, macrophage mannose receptor; C1, complement proteins 1; C2, complement protein 2.

A total of 518 proteins were less abundant in the *Aspergillus*+ BALF than in the control, among which 337 proteins were absent in the *Aspergillus*+ BALF (**Figure 1A**). Enriched gene-ontology (GO) terms are presented in **Supplementary Tables S3, S4**. Absent/less abundant proteins in the *Aspergillus*+ BALF were mainly from the innate immune function category, related to the complement system [complement activation (n=41; p=7.6x10^-4^), alternative pathway (n=12; *p*=2.8x10^-3^), classical pathway (n=43; *p*=4.2x10^-3^), and regulators of complement activation (n=17; *p*=9.6x10^-3^)] and acute-phase responses (n=14; *p*=0.012). Upregulated and downregulated humoral immune components in the *Aspergillus*+ BALF as compared to control BALF are presented in **Supplementary Table S5**. Humoral immune components that were completely absent in the *Aspergillus*+ BALF were C1q subunit-A, mannan binding lectin serine peptidase-2 (MASP2), ficolin-2 (FCN2), complement factor H related proteins 2 and 5 (CFHR2 and CFHR5), complement-8 (C8) beta-chain, *C*-type lectin receptor CD206 and surfactant protein D (SP-D). In contrast, a total of 435 proteins were more abundant in the *Aspergillus*+ BALF compared that in the control, among which 111 proteins were only identified in the *Aspergillus*+ BALF (**Figure 1B**). GO term analysis showed that the overrepresented proteins of innate immune function category in the *Aspergillus*+ BALF belong to the tumor necrosis factor-mediated signaling pathway (n=20; *p*=3.2x10^-3^), antigen processing and presentation of exogenous peptide antigen *via* MHC class I, TAP-dependent (n=20; *p*=5.4x10^-3^) and stimulatory *C*-type lectin receptor signaling pathway (n=20; *p*=0.014). While, ficolin-1 (FCN1), interleukin-8 (IL-8) and tumor necrosis factor receptor superfamily member 10C (TR10C) were detected only in *Aspergillus*+ BALF. Of note, pentraxin-3 (PTX3), CD11b and CD18 (that form complement receptor-3; CR3) were more abundant in the *Aspergillus*+ BALF (the log2 fold-changes are 5.3, 5.5, 5.1, and the *p*-values 7.6x10^-7^, 9.6x10^-8^, 1.0x10^-7^, respectively). The network of interaction between PTX3 and the complement components that are differentially expressed in the *Aspergillus*+ BALF is presented in **Figure 2**. Other humoral components more abundant in *Aspergillus*+ BALF were the antimicrobial molecules (neutrophil defensin-3, cathelicidin and galectin-10).

## Discussion

In this study, we performed a comprehensive analysis of the human BALF proteome during infection or colonization by *A. fumigatus* with a focus on the innate immune system. Regardless of the infection status (including IPA, CPA or colonization), innate immune components differentially abundant in the *Aspergillus*+ BALF majorly belonged to the humoral immune system, highlighting the implication of humoral immunity in the human lung-alveolar anti-*Aspergillus* response.

Among proteins totally absent from *Aspergillus*+ BALF, C1q, ficolin-2 and SP-D are pattern recognition molecules that have a *C*-type lectin structure and recognize microbial surfaces components as pattern recognition molecules. Ficolin-2 and SP-D can bind directly to *A. fumigatus*, interacting with β-1,3-glucan (ficolin-2) and galactomannan, galactosaminogalactan or melanin (SP-D) (9, 10). C1q knockout-mice shows increased susceptibility to IPA (11); however C1q-interactions with *A. fumigatus* is yet to be studied. *A. fumigatus* evades host complement system through binding of CFHR-1 and factor-H to the conidial surfaces (12). In the *Aspergillus*+ BALF, CFHR-1 and factor-H were found to be less abundant, whereas their structurally related components CFHR-2 and CFHR-5, however, were absent in the *Aspergillus*+ BALF. C8 and MRC1 (CD206) are the other humoral components that were absent in *Aspergillus*+ BALF. Although C8 participates in membrane attack complex, it is suggested to plays a minor role against fungal pathogens due to thickness and/or resistance of their cell wall (13). CD206, a macrophage membrane bound mannose-receptor, shows increased shedding in response to fungal pathogen (14). Absence of these proteins in the *Aspergillus*+ BALF may be due to their consumption, degradation (15) or inhibition of their biosynthesis by *A. fumigatus* and require further study.

Immune components only identified in the *Aspergillus*+ BALF were IL-8, TR10C and FCN1. While the first two are non-specific proteins of inflammatory response, the humoral component FCN1 binds to *Aspergillus* cell wall fraction and elicit the lectin pathway activation with enhanced IL-8 secretion by epithelial cells (13, 16). PTX3, an essential host protective factor and a potential diagnostic marker in *A. fumigatus* infection (17), has been reported to bind *A. fumigatus* conidia (13). FCN2 *via* crosstalk with PTX3 is believed to participate in the lectin pathway response through the recruitment of MASPs (9). Furthermore, PTX3 can directly bind C1q (18). PTX3 is importantly increased in *Aspergillus*+ BALF but C1q, FCN2 and MASP2 are completely absent, supporting a major mechanism revolving around interaction between these humoral factors. Indeed, genetic deficiency of PTX3 has been reported to contribute for the risk of *A. fumigatus* infection in those patients treated with hematopoietic stem-cell transplantation (19). This may suggest that the lack of its binding partner to execute biological function may result in the observed increased PTX3 level in the *Aspergillus+* BALF. CD11b and CD18, which form macrophage-1 antigen (complement receptor 3; CR3) that recognizes iC3b (2), were increased in the *Aspergillus*+ BALF, suggesting their shedding. Other humoral factors abundant in *Aspergillus*+ BALF were the antimicrobial molecules (neutrophil defensin-3, cathelicidin and galectin-10), may be to provide antifungal defense to some extent. Mannose-binding lectin (MBL), known to bind conidia, however, was not identified in any condition. This acute-phase protein could be absent from the control BALF and may be consumed in the *Aspergillus*+ BALF. Moreover, MBL polymorphisms were shown to be associated to aspergillosis, which cannot be ruled out in our patients from whom BALF were collected.

A recent proteome analysis of BALF was mainly focused on host and fungal protein highly expressed during IPA, but not absent/significantly less abundant proteins (20). Nevertheless, among 30 proteins found significantly increased in IPA patients in their study, our data finds agreement for 16 proteins. Discrepancies corresponded to seven proteins more abundant in control than in the *Aspergillus*+ BALF (serum amyloid protein-A, Histone H3.1, H3.2 and H2A, Apolipoprotein B-100, inter-alpha-trypsin inhibitor, lipopolysaccharide binding protein) and seven non-detected proteins. Differences observed may be due to inclusion of CPA and *A. fumigatus* colonized patients in our study. Among consistent results, seven proteins (Adipocyte plasma membrane-associated protein, vascular non-inflammatory molecule-2, carcinoembryonic antigen-related cell adhesion molecule-8, bactericidal permeability-increasing protein, dysferlin, maltase-glucoamylase and MAP-kinase 14) were detected only in the *Aspergillus*+ BALF in our analysis, and could be interesting study candidates.

A limitation of our study is the small sample size under a specific form of *Aspergillus-*related disease (IPA, CPA or colonization). Therefore, selecting the criteria of the presence or absence of *A. fumigatus* (culture, PCR, GM-Index and/or IgG level) we pooled BALF samples as control (*Aspergillus*-) and *Aspergillus*+ groups for proteomics to achieve statistical power and confidence of analyses. Humoral components showing increase or decrease may be biased upon pooling the BALF samples, as they may depend on type of aspergillosis and underlying immune status. However, we could identify several humoral components which were completely absent or present in the *Aspergillus*+ BALF compared to control, irrespective of the aspergillosis type, suggesting their role in host-*Aspergillus* interaction. We could not determine the serum level of humoral components highlighted by BALF proteomics, as respective sera were no longer available. Indeed, determining their serum levels could have helped decipher between consumption/degradation at local site of infection versus initial deficiency of these humoral components. Nevertheless, our study may pave the way to identification of new candidate proteins to better understand the pathophysiology of *A. fumigatus*. Furthermore, identifying signature proteins may contribute to improving diagnosis of aspergillosis or provide new therapeutic leads as shown with PTX3 or SP-D. Knockout mice of PTX3 and SP-D are highly susceptible to IPA, and the external intranasal administration of exogenous PTX3 or SP-D in their respective knockout mice challenged with *A. fumigatus* conidia significantly improved mice survival rates as compared to the control group of mice (10, 21).

A recent study has evaluated the cytokine levels in the BALF samples of at-risk patients for IPA, and they demonstrated a specific alveolar cytokine profile for IPA (22). Interestingly, among the 32 analytes tested in this study, IL-8 was the dominant discriminator between infected patients and uninfected individuals, predicting IPA with elevated sensitivity (90%), specificity (73%) and negative predictive value (88%). This is confirmed by our results with IL-8 being only detected in *Aspergillus*+ BALF. Chemokines are signaling molecules secreted by the immune cells, they are variable depending on pathogen and interacting immune cells. While, the diverse origin of humoral immune components may have the advantage over cytokines as the diagnostic markers, as their levels could vary because of their consumption, depletion or inhibition of their biosynthesis by invading pathogen. Exploitation of humoral immune components as the aspergillosis diagnostic markers, however, requires confirmation with categorized analysis of the IPA, CPA and colonized BALF samples to find signature components for infection versus colonization, as well as exploring other fungal, bacterial and viral infected BALF to identify *Aspergillus*-specific signature.

Overall, our data confirms/supports the importance of several humoral components of the host innate immune arm that were underexplored, and identifies humoral components that were not previously studied in the context of host-*Aspergillus* interaction. These new leads require further investigation to understand the interplay between humoral immune system of the host and *A. fumigatus*. Moreover, the humoral components that were completely absent or only present in the *Aspergillus*+ BALF could be potential immunodiagnostic markers.

## Data Availability Statement

The proteomics mass-spectrometry data have been deposited to the ProteomeXchange Consortium via the PRIDE partner repository (dataset identifier PXD023417).

## Ethics Statement

Ethical review and approval were not required for the study on human participants in accordance with the local legislation and institutional requirements. Written informed consent for participation was not required for this study in accordance with the national legislation and the institutional requirements.

## Author Contributions

VA designed and planned this study. SD, MD, and SW performed experiments. SD, MD, SW, HG, and QG analysed data. MM, J-PG, and VA evaluated data. SD and VA drafted manuscript. All authors contributed to the article and approved the submitted version.

## Funding

This work was supported by ANR-FUNHYDRO (ANR-16S-CE110020-01) grant.

## Conflict of Interest

The authors declare that the research was conducted in the absence of any commercial or financial relationships that could be construed as a potential conflict of interest.
