# Supplementary material for: Proteomic Analysis of Humoral Immune Components in Bronchoalveolar Lavage of Patients Infected or Colonized by Aspergillus fumigatus
Source: Front Immunol. 2021 May 26;12:677798. doi: 10.3389/fimmu.2021.677798 (PMC8187748; doi:10.3389/fimmu.2021.677798)
Supplement: Supplementary file 1 [file DataSheet_1.pdf]

Supplementary Material

Table S1. Characteristic of the individuals from whom broncholaveolar lavage (BALF) were collected

**Characteristics**	***Aspergillus* group (n=10)**	**Control group (n=10)**
Age (median [IQ])	61 [53-65]	61 [51-66]
M/F sex ratio	2.33	0.67
**BALF indication**		
Suspicion of cancer	1/10	2/10
Suspicion of infection	9/10	6/10
Suspicion of interstitial pneumonia	0/10	2/10
**Microbiological data**		
Mycology		
Direct BALF examination with hyphae	1/10	0/10
Positive BALF *Aspergillus* culture	10/10	0/10
Positive BALF *Aspergillus* PCR	7/10	0/10
BALF GM >1	6/10	0/10

Serum GM >0.5	0/6	0/3
Anti-*Aspergillus* antibody serology	3/8	0/3
Bacteriology		
Positive BALF culture (>10^3 CFU/mL)	0/10	0/10
Virology		
Positive PCR	0/10	2/7*
**Type of aspergillosis**		
IPA	3/10	-
CPA	3/10	-
Colonization	4/10	-

*One positive *Rhinovirus* PCR and one positive CMV PCR (2.37 log). BALF: bronchoalveolar lavage fluid; CPA: chronic pulmonary aspergillosis; GM: galactomannan; IPA: invasive pulmonary aspergillosis; IQ: interquartile; M/F: male/female.

Table S2. Characteristic of the *Aspergillus+* individuals from whom broncholaveolar lavage (BALF) were collected and pooled

Patients	No. 1	No. 2	No. 3	No. 4	No. 5	No. 6	No. 7	No. 8	No. 9	No. 10
EORTC **Host factor**	Chemotherapy	Severe influenza	None	Corticosteroid	None	None	High dose corticosteroids and lymphocyte specific monoclonal Ab	None	None	None
Underlying chronic lung disease	Lung carcinoma	None	Sarcoidosis	Asthma	COPD	Severe sarcoidosis, bi-apical fibrosis	None	Tuberculosis sequelae	None	Sarcoidosis
**Imaging**	Lobar consolidation excavated micronodule	Nodules, ground-glass opacificatin	Typical aspergilloma	Lobar consolidations excavated micronodules	Emphysema	Compatible with CPA	Not contributive	Large cavities	Pseudo-nodular condensations	Not contributive
**Mycology** BAL Direct examination	Negative	Negative	Negative	Negative	Septate hyphae	Negative	Negative	Negative	Negative	Negative
BAL culture	*Aspergillus fumigatus*									
BAL *Aspergillus* PCR	Positive (Ct=32.28)	Negative	Positive (Ct=37.7)	Negative	Positive (Ct=23.63)	Positive (Ct=30.52)	Positive (Ct=38.48)	Positive (Ct=28.70)	Positive (Ct=39.19)	Negative
GM index in BAL	>6.00	1.21	0.94	>6.00	>6.00	>6.00	0.25	>6.00	0.11	0.41
GM index in serum	0.128	0.204/0.419	0.098	0.143	NA	NA	NA	NA	0.066	NA
Aspergillus IgG	NA	NA	Positive	NA	NA	Positive	NA	Positive	NA	NA
Diagnogis	IPA	IPA (IAPA)	CPA	IPA	Colonization	CPA	Colonization	CPA	Colonization	Colonization

Ab: antibody; CPA: Chronic pulmonary aspergillosis; IAPA: Influenza-associated pulmonary aspergillosis; IPA: Invasive pulmonary aspergillosis;

NA: Not available.

Table S3. Enriched gene ontology terms (biological process) among proteins absent or significantly less abundant in *Aspergillus*+ BALF from lowest to highest *p*-value

Term	Count	*p*-Value	Fold-enrichment
GO:0010951~negative regulation of endopeptidase activity	34	2.61E-04	1.62887397
GO:0006956~complement activation	41	7.61E-04	1.49655647
GO:0001523~retinoid metabolic process	15	0.00112186	2.02904716
GO:0006957~complement activation, alternative pathway	12	0.00277809	2.12269549
GO:0006958~complement activation, classical pathway	43	0.00422972	1.39270748
GO:0019083~viral transcription	18	0.00567277	1.72469008
GO:0006412~translation	20	0.0072165	1.64256198
GO:0000184~nuclear-transcribed mRNA catabolic process, nonsense-mediated decay	20	0.0072165	1.64256198
GO:0030449~regulation of complement activation	17	0.00957622	1.69969457
GO:0006364~rRNA processing	18	0.01069457	1.65570248
GO:0006953~acute-phase response	14	0.01230611	1.78856749
GO:0006413~translational initiation	21	0.0133899	1.55778459
GO:0007597~blood coagulation, intrinsic pathway	11	0.01472268	1.9458042
GO:0006614~SRP-dependent cotranslational protein targeting to membrane	18	0.01866935	1.59202161
GO:0006508~proteolysis	62	0.02252325	1.22908948
GO:0030198~extracellular matrix organization	16	0.02775899	1.59971254
GO:0006869~lipid transport	11	0.03174372	1.80681818

GO:0060333~interferon-gamma-mediated signaling pathway	9	0.04800529	1.88148009
GO:0042157~lipoprotein metabolic process	10	0.09468566	1.64256198
GO:0044267~cellular protein metabolic process	19	0.09600334	1.36537965
GO:0001934~positive regulation of protein phosphorylation	12	0.09921292	1.53305785

Table S4. Enriched gene ontology terms (biological process) among proteins only present or significantly more abundant in *Aspergillus*+ BALF from lowest to highest *p*-value

**Term**	**Count**	***p*-Value**	**Fold-enrichment**
GO:0000165~MAPK cascade	24	0.00282076	1.68169227
GO:0033209~tumor necrosis factor-mediated signaling pathway	20	0.00320563	1.77511962
GO:0060071~Wnt signaling pathway, planar cell polarity pathway	21	0.00523007	1.69443236
GO:0000209~protein polyubiquitination	21	0.00523007	1.69443236
GO:0002479~antigen processing and presentation of exogenous peptide antigen via MHC class I, TAP-dependent	20	0.0054467	1.71785769
GO:0050852~T cell receptor signaling pathway	24	0.00696113	1.59760766
GO:0002223~stimulatory C-type lectin receptor signaling pathway	20	0.01370389	1.61374511
GO:0051436~negative regulation of ubiquitin-protein ligase activity involved in mitotic cell cycle	17	0.01645495	1.67650186
GO:0031145~anaphase-promoting complex-dependent catabolic process	17	0.01645495	1.67650186
GO:0038061~NIK/NF-kappaB signaling	17	0.01645495	1.67650186
GO:0090263~positive regulation of canonical Wnt signaling pathway	17	0.02541951	1.61662679
GO:0051437~positive regulation of ubiquitin-protein ligase activity involved in regulation of mitotic cell cycle transition	17	0.02541951	1.61662679
GO:0006521~regulation of cellular amino acid metabolic process	16	0.02721837	1.63857195
GO:0006283~transcription-coupled nucleotide-excision repair	6	0.03011223	2.66267943

GO:0071377~cellular response to glucagon stimulus	6	0.03011223	2.66267943
GO:0070527~platelet aggregation	12	0.03457016	1.77511962
GO:0046686~response to cadmium ion	7	0.03533982	2.3298445
GO:0007229~integrin-mediated signaling pathway	10	0.03736643	1.90191388
GO:0006936~muscle contraction	9	0.03797086	1.99700957
GO:0051092~positive regulation of NF-kappaB transcription factor activity	14	0.0474753	1.62076139
GO:0090090~negative regulation of canonical Wnt signaling pathway	18	0.0490876	1.49775718
GO:0043488~regulation of mRNA stability	21	0.05152358	1.43375046
GO:0035556~intracellular signal transduction	12	0.05507303	1.68169227
GO:0007568~aging	15	0.06350819	1.53616121
GO:0010800~positive regulation of peptidyl-threonine phosphorylation	5	0.06854783	2.66267943
GO:0043406~positive regulation of MAP kinase activity	5	0.06854783	2.66267943
GO:0043087~regulation of GTPase activity	5	0.06854783	2.66267943
GO:0042554~superoxide anion generation	5	0.06854783	2.66267943
GO:0030220~platelet formation	5	0.06854783	2.66267943
GO:0045730~respiratory burst	5	0.06854783	2.66267943
GO:0006909~phagocytosis	8	0.07049263	1.93649413
GO:0050832~defense response to fungus	7	0.07291433	2.07097289
GO:0006098~pentose-phosphate shunt	7	0.07291433	2.07097289
GO:0042102~positive regulation of T cell proliferation	7	0.07291433	2.07097289
GO:0071363~cellular response to growth factor stimulus	6	0.07313093	2.28229665

GO:0050900~leukocyte migration	13	0.07547376	1.57340148
GO:0007165~signal transduction	39	0.07979452	1.23624402
GO:0043161~proteasome-mediated ubiquitin-dependent protein catabolic process	19	0.0815594	1.40530303
GO:0051603~proteolysis involved in cellular protein catabolic process	15	0.08810032	1.47926635
GO:0043066~negative regulation of apoptotic process	26	0.08962802	1.3062201

Table S5. Humoral immune components upregulated and downregulated in *Aspergillus*+ BALF compared to control BALF

Uniprot ID	Protein name	log2 [Mean in Control] [Mean in Infected	*p*-Value
Downregulated humoral immune components (More abundant in control BALF than in *Aspergillus*+ BALF)			
P02746	Complement C1q subcomponent subunit B	4,770011924	3,95752E-08
P02747	Complement C1q subcomponent subunit C	4,605702107	1,60372E-08
P00746	Complement factor D	4,194292887	5,94645E-07
P05155	Plasma protease C1 inhibitor	4,140421559	2,59592E-07
P09382	Galectin-1	4,105476523	1,15874E-06
A0A0B4J1V2	Immunoglobulin heavy variable 2-26	3,764981179	2,34301E-06
P02748	Complement component C9	3,627810854	6,92308E-05
P0DJI8	Serum amyloid A-1 protein	3,395388246	3,14203E-06
P13671	Complement component C6	3,352172164	1,47994E-07
P0C0L5	Complement C4-B	3,15275932	1,52395E-05
P0DJI9	Serum amyloid A-2 protein	3,149796889	4,41139E-07
A0A075B6R2	Immunoglobulin heavy variable 4-4	3,108093718	6,82994E-06
P06681	Complement C2	3,040113337	1,91103E-05
O75636	Ficolin-3	2,916692071	1,08101E-05
A0A0A0MS15	Immunoglobulin heavy variable 3-49	2,739836513	4,07208E-05

P07357	Complement component C8	2,682195668	1,46374E-06
P00736	Complement C1r subcomponent	2,643074991	1,92725E-05
P07988	Pulmonary surfactant-associated protein B	2,640060845	3,07028E-05
P10643	Complement component C7	2,542900684	3,52392E-05
P05156	Complement factor I	2,488885136	8,72817E-06
P01817	Immunoglobulin heavy variable 2-5	2,371645737	0,001213779
P01031	Complement C5	2,365291696	1,17243E-05
P09871	Complement C1s subcomponent	2,299412154	5,86885E-06
P00751	Complement factor B	2,188312871	1,62463E-05
P04430	Immunoglobulin kappa variable 1-16	2,10389612	5,86363E-06
P02741	C-reactive protein	2,089017067	8,64115E-06
P01859	Immunoglobulin heavy constant gamma 2	2,027420903	1,92991E-05
P07360	Complement component C8 gamma chain	2,002651275	6,96087E-05
A0A0B4J1V0	Immunoglobulin heavy variable 3-15	1,993099531	0,002899603
P08603	Complement factor H	1,932699629	6,12734E-06
A0A0B4J1Y9	Immunoglobulin heavy variable 3-72	1,866696824	0,000161255
P04003	C4b-binding protein alpha chain	1,684753798	5,38417E-05
P0C0L4	Complement C4-A	1,612493675	1,58024E-05
A0M8Q6	Immunoglobulin lambda constant 7	1,579869075	0,03020627
P01701	Immunoglobulin lambda variable 1-51	1,577387914	0,082752851
A0A0J9YX35	Immunoglobulin heavy variable 3-64D	1,516585817	6,21991E-05

P20851	C4b-binding protein beta chain	1,44145692	0,002047169
Q08380	Galectin-3-binding protein	1,340356215	0,000280963
Q03591	Complement factor H-related protein 1	1,306652542	0,000509482
P15814	Immunoglobulin lambda-like polypeptide 1	1,237977737	0,00223212
P01024	Complement C3	1,166748475	0,000183013
A0A0B4J2H0	Immunoglobulin heavy variable 1-69D	1,053094216	0,003030123
P17931	Galectin-3	1,015221387	0,009673571
P01619	Immunoglobulin kappa variable 3-20	1,010094094	0,001695191
Upregulated humoral immune components (More abundant in *Aspergillus*+ BALF than in control BALF)			
P05107	Integrin beta-2 (CD18)	-5,60480845	4,79884E-07
P11215	Integrin alpha-M (CD11b)	-5,528917834	4,24283E-07
P26022	Pentraxin-related protein PTX3	-5,286034363	7,60483E-07
P49913	Cathelicidin antimicrobial peptide	-4,743953411	4,37255E-07
Q8N6C8	Leukocyte immunoglobulin-like receptor subfamily A member 3	-3,597574612	1,44304E-05
Q05315	Galectin-10	-3,509452792	3,62799E-06
O75015	Low affinity immunoglobulin gamma Fc region receptor III-B	-3,241484206	5,60146E-06
Q9NPY3	Complement component C1q receptor	-3,239587341	1,41198E-05

P08174	Complement decay-accelerating factor	-2,417185615	0,000699514
P01591	Immunoglobulin J chain	-2,075119788	6,40722E-06
P13987	CD59 glycoprotein	-2,067521095	4,00212E-05
P01833	Polymeric immunoglobulin receptor	-2,059819297	5,42548E-06
P01877	Immunoglobulin heavy constant alpha 2	-1,976464062	0,000331039
P59666	Neutrophil defensin 3 (DEF3)	-1,618669146	0,000148637
A0A075B6I0	Immunoglobulin lambda variable 8-61	-1,177521776	0,000758058
P01861	Immunoglobulin heavy constant gamma 4	-1,118881252	0,000523796
A0A0B4J1V6	Immunoglobulin heavy variable 3-73	-1,003499456	0,002879841

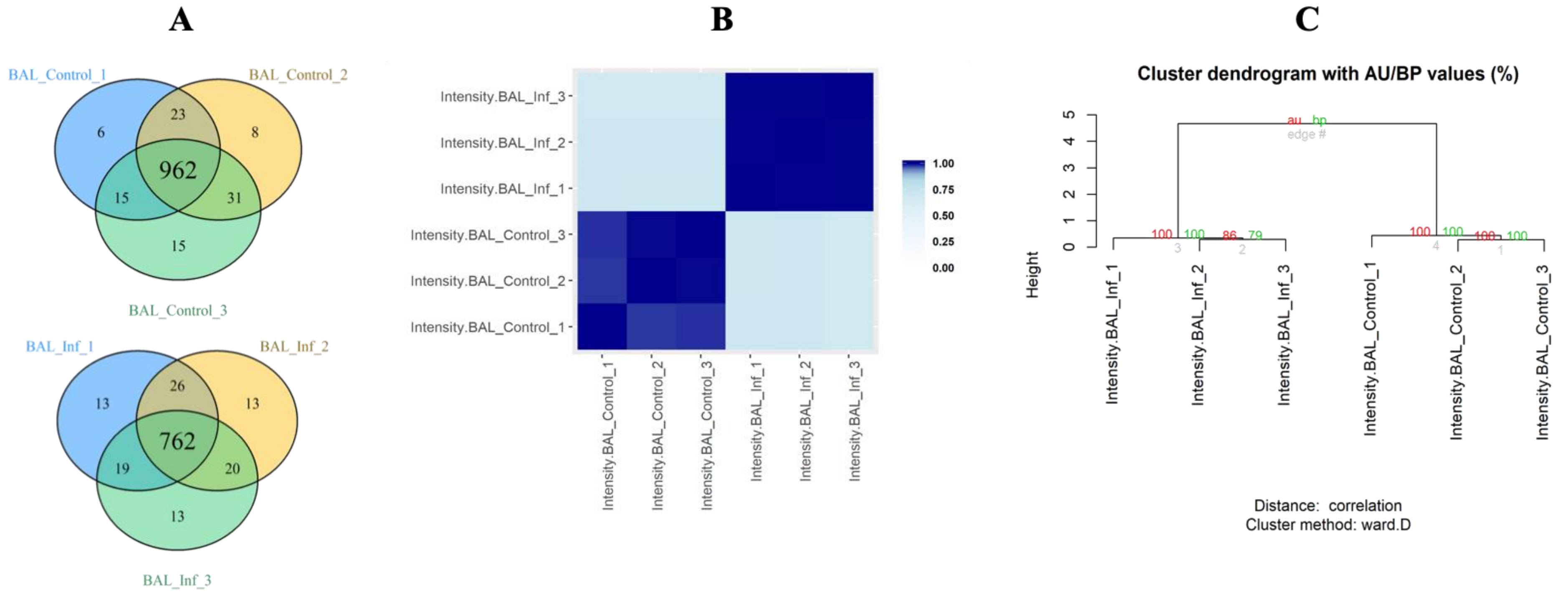

Figure S1. Number of identified protein and correlation between replicates. Venn diagram with number of identified proteins by replicate after deleting reverse, contaminants and only identified by site protein in (A) control bronchoalveolar lavage (BALF) and *Aspergillus*+ BALF. (B) Correlation matrix representing correlation between each pair of samples computed using all complete pairs of observation in the samples. (C) Cluster dendrogram showing approximately unbiased (AU) probability value and bootstrap probability (BP) value corresponding to the frequency of proteins identified in the technical replicates.
